# Pharmacokinetic Study of Di-Phenyl-Di-(2,4-Difluobenzohydroxamato)Tin(IV): Novel Metal-Based Complex with Promising Antitumor Potential

**DOI:** 10.1155/2012/210682

**Published:** 2012-02-16

**Authors:** Yunlan Li, Zhuyan Gao, Pu Guo, Qingshan Li

**Affiliations:** School of Pharmaceutical Science, Shanxi Medical University, Taiyuan 030001, China

## Abstract

Di-phenyl-di-(2,4-difluobenzohydroxamato)tin(IV)(DPDFT), a new metal-based arylhydroxamate antitumor complex, showed high *in vivo* and *in vitro* antitumor activity with relative low toxicity, but no data was reported regarding its pharmacokinetics and dependent toxicity. In this paper, a rapid, sensitive, and reproducible HPLC method *in vivo* using Diamonsil ODS column with a mixture of methanol and phosphoric acid in water (30 : 70, V/V, pH 3.0) as mobile phase was developed and validated for the determination of DPDFT. The plasma was deproteinized with methanol that contained acetanilide as the internal standard (I.S.). The photodiode array detector was set at a wavelength of 228 nm at room temperature and a linear curve over the concentration range 0.1~25 *μ*g*·*mL^−1^ (*r* = 0.9993) was obtained. The method was used to determine the concentration-time profiles for DPDFT in the plasma after single intravenous administration with doses of 5, 10, 15 mg*·*kg^−1^ to rats. The pharmacokinetics parameter calculations and modeling were carried out using the 3p97 software. The results showed that the concentration-time curves of DPDFT in rat plasma could be fitted to two-compartment model.

## 1. Introduction

Metals offer potential advantages over the more common organic-based drugs, including a wide range of coordination numbers and geometries, accessible redox states, “tuneability” of the kinetics of ligand substitution, as well as a wide structural diversity. Medicinal inorganic chemistry is a thriving area of research [1, 2], initially fueled by the discovery of cisplatin, a metal-based antitumor drug about 40 years ago. Since the discovery of cisplatin and its introduction in the clinics, metal compounds have been intensely investigated in view of their possible application in cancer therapy. Platinum anticancer agents, such as cisplatin, have been highly successful but there are several disadvantages associated with their clinical use. What needs to be recognized is that there are many other nonplatin metal-based antitumor drugs in the periodic table with therapeutic potential. Diorganotin(IV) complexes are potential antitumor agents mainly active against P388 lymphocytic leukemia and other tumors [3–5]. Lately, these antitumor agents are actually being studied widely. Among diorganotin(IV) compounds, dibutyltin(IV) derivatives of hydroxamic acid have received more attention due to their structural and biological importance [6–8].

**Figure 1:**
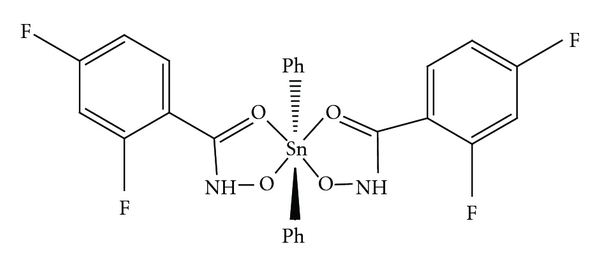
Chemical structure of DPDFT.

Recently, we reported a series of diorganotin(IV) arylhydroxamates which exhibit *in vitro* antitumor activities (against a series of human tumor cell lines) which, in some case, are identical to, or even higher than, that of *cisplatin *[3, 5]. Di-phenyl-di-(2,4- difluobenzohydroxamato)tin(IV) (DPDFT, its structure was shown in Figure 1), a kind of efficient diorganotin(IV) patent compound (number: ZL01135148 and 102826Z) with lower toxicity, is a potential antitumor candidate for the clinical application due to its high *in vivo and in vitro* activity mainly against hepatoma, gastric cancer, nasopharyngeal carcinoma and other tumors (data not shown here). However, its antitumor molecular action mechanism is still unclear. In order to study the precise action mechanism and toxicity of this metal-based antitumor diorganotin(IV) compound, its fate should be first elucidated *in vivo*, including their absorption, distribution, metabolism, and elimination. However, nothing is known about the pharmacokinetic behavior of DPDFT in body. Therefore, it is essential to establish a rapid, sensitive, and accurate method to determine the pharmacokinetic of organotin compound DPDFT in a body. 

For medical care, practical, robust, simple, and efficient analytical methods are needed. Several methods have been reported for the determination of organotin compounds, including HPLC-MS [9, 10], HPLC-ICP-MS [10, 11], GC [12], GC-MIP AED [12–14], GC-MS [15–17], GC-ICP MS [18, 19], and so on. However, these methods have obvious disadvantages. For examples, GC methods have complicated pretreatments for the samples and seem to be unsuitable for quantitative determination in rat plasma because only a small amount of blood is normally used in pharmacokinetic studies. HPLC-MS is superior method and should be used whenever is possible. MS methods were used for mentioned analysis in order to increase selectivity of detection of DPDFT from complex matrixes. However, in this study, the HPLC-MS method was not used because the mass spectrometry conditions were still not ripe for DPDFT detection, and the high analysis cost and the expensive apparatus required were other considerations. So far, to the best of our knowledge, no method has been reported for determination of the diorganotin(IV) patent compound DPDFT by HPLC method with UV detection in the pharmacokinetic studies in rat plasma. Therefore, in this research, we chose DPDFT as a typical antitumor diorganotin(IV) arylhydroxamate to develop a simple, sensitive, and specific HPLC assay for its quantitative determination in rat plasma and to investigate its preliminary pharmacokinetic properties.

## 2. Experimental

### 2.1. Materials and Reagents

DPDFT used in analysis was synthesized by the same method as described in [20]. Its purity was 99.9%. Acetanilide used as an internal standard (I.S.) was purchased from Sigma Laboratories (St. Louis, MO). The chromatographic solvents and reagents such as methanol and phosphoric acid were obtained from the National Institute for the Control of Pharmaceutical and Biologic Products (Beijing, China). All substances were of chromatographic grade. Deionized water was prepared using a Milli-Q water purifying system from Millipore Corp. (Bedford, MA).

### 2.2. Animal Treatment

Laboratory bred adult Wistar albino rats (200–250 g), which were supplied by the Animal Research Center at Shanxi Medical University (Taiyuan, Shanxi Province, China), were housed at 25 ± 2°C in a well-ventilated animal house under 12 : 12 h light dark cycle. The animals drank sterilized drinking water, and standard chow diet was supplied *ad libitum* to each cage. The animal experiments were performed in accordance with the ARVO Statement for the Use of Animals in Ophthalmic and Vision Research and were approved by the Animal Ethics Committee of Shanxi Medical University.

### 2.3. Instrumentation and Chromatographic Conditions

HPLC analysis was carried out using a Waters 2695 HPLC system (Waters Associates, Milford, MA) which consisted of a photodiode array detector, an autosampler, and a degasser. The apparatus was interfaced to a DELL PC compatible computer using Empower Pro software for data acquisition. The sensitivity was 0.2 AUFS. The autosampler was cooled to 10°C. The column was maintained at room temperature. Chromatographic separation of DPDFT and the I.S. was achieved on a Diamonsil C_18_ column (250 mm × 4.6 mm, 5 *μ*m) from Dikma Technologies (Beijing, China) protected by a SHIMADZU LC guard column at 25°C. The mobile phase for HPLC analysis consisted of phosphoric acid in water (solvent A)/methanol (solvent B) (30 : 70, V/V, pH 3.0) with a flow rate of 0.8 mL·min^−1^. A sample volume of 20 *μ*L was injected. Prior to use, the mobile phase was filtered through a 0.45 *μ*m hydrophilic membrane filter. The photodiode array detector was set at a wavelength of 228 nm at room temperature. Under the chromatographic condition mentioned above, DPDFT and the I.S. acetanilide could be separated completely in the chromatograms (*R* > 1.5), and there was no endogenous interference with the chromatographic peak of DPDFT and the I.S.acetanilide. Besides, the retention time of acetanilide (*t*_*R*_ = 15.67 min) was very suitable as the I.S. compared to that of DPDFT (*t*_*R*_ = 8.34 min).

### 2.4. Preparation of Plasma Samples

Blood samples collected from rat blood plasma were immediately transferred to 1.5 mL heparinized microcentrifuge tubes from fossa orbitalis of rats, and then processed for plasma by centrifugation. The supernatant plasma (0.2 mL) was then vortex-mixed with methanol (0.4 mL) containing acetanilide (0.2 mL, 50.0 *μ*g·mL^−1^) as internal standard (I.S.) for 30 s. After vortex-mixing, the mixture was centrifuged at 13000 rpm for 10 min 4°C to separate precipitated proteins. The supernatant solution of methanol layer was filtered through a 0.45 *μ*m membrane filter. Twenty microliters of filtrate were injected into the chromatography. The same sample processing was also applied to the recovery and to the precision study in plasma. All harvested samples stored at −4°C were brought to room temperature before use and analyzed within one week. No significant differences were found between the samples stored at −4°C and those stored at −20°C (data not shown).

### 2.5. Bioanalytical Method Validation

#### 2.5.1. Preparation of Stocks, Calibration Standards, and Quality Control Samples

A stock solution of DPDFT was prepared in methanol at the concentration of 100 mg·mL^−1^ and was further diluted in HPLC mobile phase to make working standards. The I.S. stock solution was prepared and diluted to 50.0 *μ*g·mL^−1^ working solution with HPLC mobile phase. All the stock solutions were maintained at 4°C until use.

The linearity of HPLC method for the determination of DPDFT was evaluated by a calibration curve in the range of 0.1~25 *μ*g·mL^−1^. Calibration standard samples were prepared by adding different concentrations of the standards of DPDFT and 0.2 mL of the I.S. working solution to the blank plasma. The final concentrations of DPDFT standard samples of plasma (0.1, 0.5, 1.0, 2.5, 5.0, 10.0, and 25.0 *μ*g·mL^−1^, resp.) were prepared by spiking control rat plasma with appropriate amounts of the standard stock solution prepared above. The I.S. was added to each standard sample immediately before sample processing. For the evaluation of the linearity of the standard calibration curve, the analyses of DPDFT in plasma samples were performed on three independent days using fresh preparations. Each calibration curve consisted of a double blank sample (without internal standard), a blank sample (with internal standard), and seven calibrator concentrations. Each calibration curve was constructed by plotting the analyte to internal standard peak area ratio (*y*) against analyte concentrations (*x*). The calibration curves were fitted using a least-square linear regression model *y* = *ax* + *b*. The resulting *a*, *b* parameters were used to determine back-calculated concentrations, which were then statistically evaluated. All calibration curves of DPDFT were constructed before the experiments with correlation coefficient (*r* ^2^) of 0.99 or better.

#### 2.5.2. Bioanalytical Method Validation

The specificity was defined as non-interference when DPDFT was being retained from the endogenous plasma components, and no crossinterference between DPDFT and the I.S. using the proposed extraction procedure. Six different lots of blank (DPDFT-free plasma) were evaluated both with and without internal standard to assess the specificity of the method.

Quality control (QC) samples were used to determine the accuracy and precision of method and were independently prepared at low (0.8 *μ*g·mL^−1^), medium (4 *μ*g·mL^−1^), and high (20 *μ*g·mL^−1^) concentrations. To evaluate the accuracy and precision, we used at least five QC samples of three different concentrations of DPDFT. The intraday and interday accuracies were expressed as the percentage difference between the measured concentration and the nominal concentration in rat plasma. The intraday precision and accuracy were calculated using replicate (*n* = 6) determinations for each concentration of the spiked plasma sample during a single analytical run. The interassay precision and accuracy were calculated using replicate (*n* = 6) determinations of each concentration made on three separate days. The variability of determination was expressed as the relative standard deviation (RSD) which should be ≤15%, covering the range of actual experimental concentrations.

The extraction efficiency of DPDFT was determined by analyzing replicate sets (*n* = 6) of QC samples: 0.4, 8, 20 *μ*g·mL^−1^ for rat plasma, representing low, medium, and high QCs, respectively. The recoveries were calculated by comparing the peak areas of DPDFT added into blank samples and extracted using the protein precipitation procedure, with those obtained from DPDFT spiked directly into postprotein precipitation solvent at three QC concentration levels.

The stability of DPDFT in rat plasma was assessed by analyzing replicates (*n* = 6) of QC samples at concentrations of 0.8, 4, 20 *μ*g·mL^−1^, respectively. The investigation covered the expected conditions during all of the sample storage and process periods, which included the stability data from freeze/thaw cycle and long-term stability tests. The concentrations obtained from stability studies were compared with the freshly prepared QC samples, and the percentage concentration deviation was calculated. In each freeze–thaw cycle, the samples were frozen and stored at −20°C for 10 days, then thawed at room temperature. The stability of the fresh plasma samples was tested after keeping the samples at −4, −20°C and room temperature for 72 h. The stability of deproteinized samples at 10°C in the autosampler was evaluated up to 24 h.

To determine the limit and quantification of detection, we prepared the dilutions of 1, 2.5, 5, 10, 15, 20, and 30 ng·mL^−1^ DPDFT in plasma. The results were evaluated by analyzing each plasma sample spiked with the analyte at a final concentration at which the signal-to-noise ratio (S/N) was 10 and 3.

#### 2.5.3. Pharmacokinetic Study

To evaluate the suitability of the assay for pharmacokinetic studies, 7.5, 15, and 30 mg·kg^−1^ of DPDFT were intravenously administered to rats (half males and half females, resp.). Six animals were used in each dosage by direct injection into a lateral tail vein, with a duration of infusion less than 1 min. Heparinized blood samples (0.5 mL) were collected at 0, 1, 3, 5, 10, 30, 60, and 120 min after injection. Eighteen rats were used for each time point. After each sampling, the removed volume of blood was supplemented with an equal volume of sodium chloride. The blood samples were immediately centrifuged and the resulting plasma was prepared according to the procedure given for the calibrators. Pharmacokinetic calculations were performed using the observed data. Pharmacokinetic analysis of DPDFT concentrations in plasma was performed using two-compartment model methods via the 3p97 software package (Chinese Pharmacology Society). All values obtained were expressed in mean ± standard deviation.

## 3. Results and Discussion

A deproteinized method to detect DPDFT, an typical antitumor diorganotin(IV) compound in plasma, was first developed in this paper. Diverse proportional solvents (methanol and acidified water) were selected in the deproteinizing process. The plasma deproteinized with double volume of methanol could produce the minimal dilution, optimum peak shape, and an increase of detector sensitivity along with the satisfactory recovery.

### 3.1. Wavelength Selection Results

**Figure 2:**
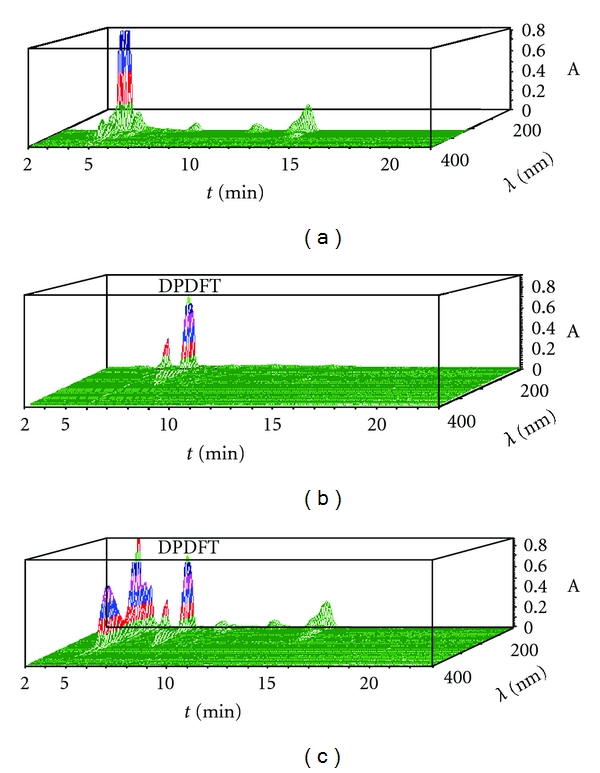
HPLC-DAD profiles of DPDFT in plasma sample. (a) Blank plasma; (b) DPDFT (2 *μ*g·mL^−1^) standard; (c) blood sample containing DPDFT (2.4 *μ*g·mL^−1^) collected at 3 min after administration of DPDFT (15 mg·kg^−1^, i.v.).

The Waters 2695 HPLC–DAD measurement was performed under the chromatographic condition mentioned above. Chromatographic separation of DPDFT was achieved on a Diamonsil C_18_ column (250 mm × 4.6 mm, 5 *μ*m) protected by a SHIMADZU guard column at 25°C. The mobile phase for HPLC analysis consisted of phosphoric acid in water (solvent A)/methanol (solvent B) (30 : 70, V/V, pH 3.0) with a flow rate of 0.8 mL·min^−1^. A sample volume of 20 *μ*L was injected. The DAD wavelength range was set on 190~400 nm with a slid width of 1 nm and a response time of 2.0 s at room temperature. As shown in Figures 2(a), 2(b), and 2(c), the peaks were detected with good baseline separation. Peak identification was confirmed by comparison of UV spectra. The maximum absorption wavelength of DPDFT was 228 nm, and there was no endogenous interference with the chromatographic peak of DPDFT.

### 3.2. Validation Data of Bioanalytical Method

The method was validated using the criteria described above. The data were found to be linear over a concentration range of 0.1~25 *μ*g·mL^−1^ in blood samples. The regression equation was *y* = 32.001*x* + 0.31, with the correlation coefficient *r* = 0.9993 (*n* = 7), where y represented the peak-area ratio of DPDFT to the I.S. in rat plasma and x was the concentration of DPDFT. The limit of quantitation (LOQ) was 10 ng, which can be determined with a relative error (RE) and precision (RSD) of <15% at a signal to-noise ratio of 10. The limits of detection (LOD) were 3.5 ng, based on a signal-to-noise ratio of 3.

**Figure 3:**
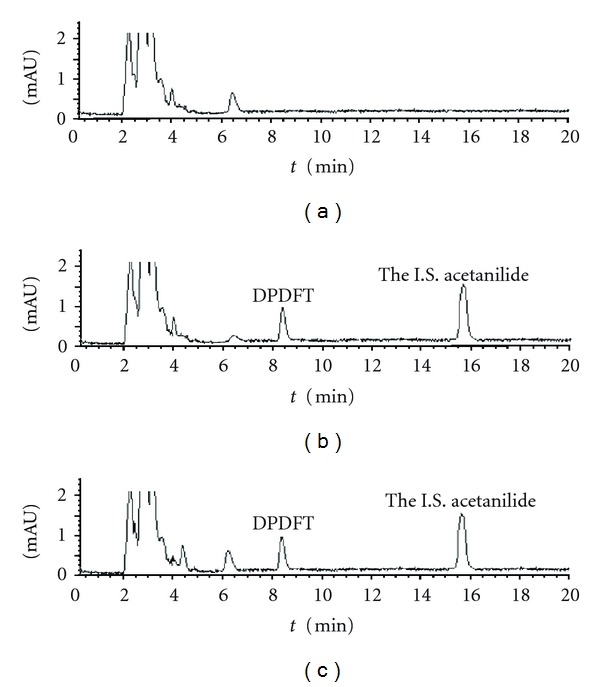
Chromatograms of DPDFT in plasma sample. Separation was performed using Waters 2695 HPLC system. The mobile phase consisted of phosphoric acid in water (solvent A)/methanol (solvent B) (30 : 70, V/V, pH 3.0) using Diamonsil C_18_ column at 25°C with a flow rate of 0.8 mL·min^−1^. (a) Blank plasma; (b) blank plasma spiked with DPDFT (3 *μ*g·mL^−1^) and the I.S. (8 *μ*g·mL^−1^); (c) blood sample containing DPDFT (2.4 *μ*g·mL^−1^) collected at 3 min after administration of DPDFT (15 mg·kg^−1^, i.v.).

Under the chromatographic condition, the number of theoretical plates was 5000. The degree of interference by endogenous plasma with DPDFT and the I.S. was assessed by inspection of chromatograms derived from a processed blank plasma sample. The results show that there were no endogenous interfering peaks with the I.S. and DPDFT in the rat plasma. Typical chromatograms of blank plasma, blank plasma spiked with DPDFT QC sample (3 *μ*g·mL^−1^) and the I.S., and a rat plasma sample after dosing with 15 mg·kg^−1^ DPDFT are presented (Figure 3). DPDFT and the I.S. were eluted at 15.67 and 8.34 min, respectively. The total run time was less than 30 min. A good separation of the I.S. and DPDFT was obtained under the specified chromatographic conditions.

The recoveries of the assay were assessed by comparing the peak-area ratios (analyte/the I.S.) obtained from spiked plasma samples of three DPDFT standard concentrations (0.4, 8, 20 mg/mL) with the peak-area ratios (analyte/the I.S.) for the samples containing the equivalent analyte and the I.S. which were directly dissolved in methanol. The recoveries were approximately 90.0–97.0% in the rat plasma, as shown in Table 1, the mean extraction recovery and the coefficient of variation RSD of DPDFT at three various concentrations from the rat plasma were 94.2% ± 10.3% and 10.9% (*n* = 18), respectively.

The precision and accuracy of this method were evaluated by assaying each low, middle, and high concentration QC sample. The reproducibility of the method was assessed by examining both intraday and interday variance. Accuracy (%) = [(Cobs − Cnom)/Cnom] × 100. The precision (%RSD) was calculated from the observed concentrations as follows: RSD = [standard deviation (SD)/Cobs] ×100. As shown in Table 2, the data showed that the intraday and interday precisions (% RSD) of the three QC samples in rat plasma were <15%. The RSD values of the intraday and the interday for rat plasma samples ranged from 3.8%~9.0% and 4.0%~9.0%, respectively. These validations demonstrated the reliability of the assay.

Stability of DPDFT during storage and processing was checked using quality control samples. The DPDFT and I.S. stock solutions were stable for at least 2 months when stored at 4°C. The deviation of the mean test responses was within ±10% of appropriate controls in all stability tests of DPDFT in rat plasma. After three freeze-thaw cycles, the concentration changes of DPDFT were less than 7%. The analyte was stable in the matrices at 4, −20°C and room temperature for 72 h without significant degradation (<8%). The run-time stability study showed that DPDFT in deproteinized rat plasma was stable at 10°C for up to 24 h (<6%). These results suggested that the rat plasma samples containing DPDFT can be handled under normal laboratory conditions without significant loss of the compound. All stability results are summarized in Table 3.

### 3.3. Pharmacokinetic Applicability

**Figure 4:**
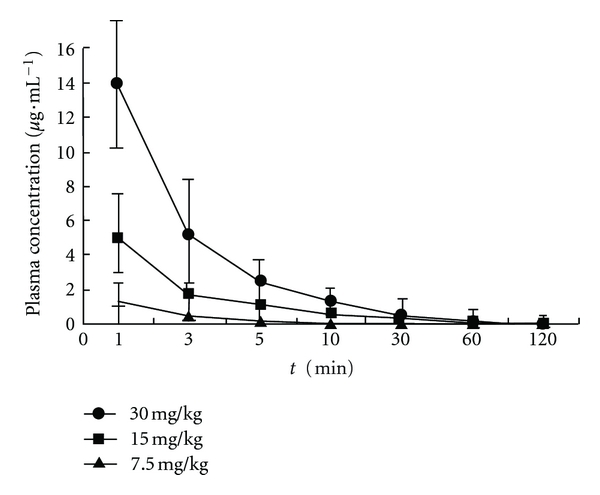
Mean plasma concentration-time profiles of DPDFT in rats after intravenous administration of 7.5, 15, and 30 mg·kg^−1^. Each point represents the mean concentration of six rats.

The developed RP-HPLC analytical method has been successfully used for the pharmacokinetic study after a single intravenous administration of DPDFT within 120 min. The mean plasma concentration-time curves of DPDFT after administration of 7.5, 15, and 30 mg·kg^−1^ in rats are shown in Figure 4, the concentration-time data conformed to a two-compartment model and the major mean pharmacokinetic parameters (mean ± SD) are summarized in Table 4. The RP-HPLC method satisfied the requirement of this study and demonstrated its general suitability for pharmacokinetics studies of DPDFT in rats.

### 3.4. The Pharmacokinetics Features of DPDFT in Rat Plasma

HPLC analysis for amphoteric, polar substances with low wavelength ultraviolet absorption is always associated with some difficulties, especially when high sensitivity is required. The results showed that this classic liquid extraction HPLC with UV detecting method was sensitive (the limit of detection was 3.5 ng) enough for pharmacokinetics studies of DPDFT in rats and did not require any forms of analyte derivatization or special columns or instruments. The most important factor for achievement of high sensitivity was the clean baseline owing to appropriate sample preparation on the chromatograms. Thus, a high signal/noise ratio was achieved, which constituted the base for the high sensitivity. The second factor was the high recovery of DPDFT from plasma during the extraction process. Methanol was used not only for deproteination, but also as extractive solvent for assay. So, the high recoveries of DFDPT (90.8, 95.7, and 96.2% for three determinations, resp.) were obtained. During the process of method development, it was discovered that DPDFT spiked with aqueous I.S. showed a symmetric single peak in the chromatograms at a pH around 3.0. With high sensitivity, small sample requirement, and simple sample treatment procedures, this method was successfully applied to the analysis of rat plasma samples and the pharmacokinetic study of DPDFT in rat.

The mean pharmacokinetic parameters (mean ± SD) are summarized in Table 4. There were no significant differences in all pharmacokinetic parameters between male and female rats at dose of 7.5, 15, and 30 mg·kg^−1^. The results showed that there was significant difference for AUC(0–t), they were calculated to be 7.56, 37.15, 81.25 mg·kg^−1^·min^−1^ at doses of 7.5, 15, and 30 mg·kg^−1^, respectively, and for the value of *V*_*d*_ after three dosages, 2.13, 1.13, 0.62 L·kg^−1^, respectively. These results suggested that the pharmacokinetics of the complex is a nonlinear process from 7.5 to 30 mg/kg. Otherwise, the distribution half-life *t*_1/2*a*_ (1.04, 1.01, 1.12 min, resp.) and elimination half-life *t*_1/2*β*_ (17.68, 19.38, 16.81 min, resp.) have no significant difference when the administration dosage of DPDFT was increased from 7.5 to 30 mg/kg, indicating that DPDFT distributed and eliminated very quickly.

## 4. Conclusions

In this paper, a simple, economical, sensitive, and specific method for the determination of DPDFT, a typical antitumor diorganotin(IV) compound in rat plasma, was first reported. The assay was validated for linearity, specificity, accuracy, precision, recovery, and stability, and good results were obtained. The results of preliminary pharmacokinetic studies indicated that DPDFT showed nonlinear pharmacokinetics in the studied dose ranges in rats and the concentration-time curves of DPDFT in rat plasma could be fitted to two-compartment model. These results hinted that DPDFT might accumulate in certain organs, thus produce the toxicity or could be quickly metabolized in the plasma into active constituent for antitumor. In order to study the precise toxicity mechanism of this metal-based antitumor diorganotin(IV) compounds, we should further elucidate their *in vivo* absorption, distribution, metabolism, and elimination. Meanwhile, based on the structure of DPDFT as a lead compound, structure reconstitution and optimization should be carried out to explore the better antitumor diorganotin(IV) compounds with higher activity, relative lower toxicity, and good pharmacokinetics features in the future.

## Figures and Tables

**Table 1:** Recoveries of the assay for determining DPDFT in rat plasma (*n* = 6).

Spiked concentration (*μ*g/mL)	Recovery (%, mean ± SD)	RSD (%)
0.8	90.8 ± 7.0	7.7
4	95.7 ± 12.9	13.5
20	96.2 ± 10.8	11.3

**Table 2:** Intra- and interday precision and accuracy for DPDFT in rat plasma (*n* = 6).

Matrix	Nominal concentration (*μ*g·mL^−1^)	Observed concentration (*μ*g·mL^−1^) ± SD	Precision (%RSD)	Accuracy (%)
Intra-day	0.8	0.73 ± 0.05	6.8	−8.75
	4	3.73 ± 0.14	3.8	−6.75
	20	19.21 ± 1.02	5.3	−3.95
Inter-day	0.8	0.69 ± 0.06	8.7	−13.75
	4	3.80 ± 0.15	4.0	−5.00
	20	19.02 ± 1.11	5.9	−4.90

Notes: accuracy (%) = [(Cobs − Cnom)/Cnom] × 100, RSD = [standard deviation (SD)/Cobs] × 100.

**Table 3:** Stability results of DPDFT at different conditions in rat plasma (*n* = 6).

Storage period and storage condition	Nominal concentration (*μ*g·mL^−1^)	Observed concentration (*μ*g·mL^−1^) ± SD	Accuracy (%)	RSD (%)
Concentration of fresh preparation	0.8	0.85 ± 0.07	6.25	8.2
	4	3.91 ± 0.12	−2.25	3.1
	20	21.06 ± 1.33	5.30	6.3

Three freeze and thaw cycles	0.8	0.76 ± 0.05	−5.00	6.6
	4	3.79 ± 0.13	−5.25	3.5
	20	18.97 ± 1.17	−5.15	6.2

Stability for 72 h at 4°C	0.8	0.87 ± 0.04	8.75	4.6
	4	3.70 ± 0.16	−7.50	4.3
	20	21.14 ± 1.27	5.70	6.0

Stability for 72 h at −20°C	0.8	0.74 ± 0.05	−7.50	6.8
	4	4.22 ± 0.12	5.50	2.9
	20	19.13 ± 1.53	−4.35	7.9

Stability for 72 h at room temperature	0.8	0.78 ± 0.05	−2.50	6.4
	4	3.87 ± 0.19	−3.25	4.9
	20	19.46 ± 1.19	−2.75	6.1

Autosampler stability for 24 h at 10°C	0.8	0.83 ± 0.04	3.75	4.8
	4	3.83 ± 0.18	−4.25	4.7
	20	19.19 ± 1.08	−4.05	5.6

Notes: accuracy (%) = [(Cobs − Cnom)/Cnom] × 100, RSD = [standard deviation (SD)/Cobs] × 100.

**Table 4:** Mean pharmacokinetic parameters in rats after intravenous administration of 7.5, 15, and 30 mg·kg^−1^ of DPDFT (mean ± SD, *n* = 6).

Parameter (unit)	Dosage/mg kg^−1^		
	7.5 (low)	15 (middle)	30 (high)
*A*/min^−1^	2.21 ± 0.03	7.94 ± 3.12	22.36 ± 8.12
*B*/mg*·*kg^−1^	0.15 ± 0.04	0.92 ± 0.04	1.85 ± 0.44
**β**/min^−1^	0.04 ± 0.003	0.04 ± 0.003	0.04 ± 0.003
*V*_*d*_/L*·*kg^−1^	2.13 ± 0.07	1.13 ± 0.08	0.62 ± 0.08
*t*_1/2*a*_/min	1.04 ± 0.01	1.01 ± 0.01	1.12 ± 0.1
*t*_1/2*β*_/min	17.68 ± 2.6	19.38 ± 3.6	16.81 ± 3.6
*K*_21_/min^−1^	0.08 ± 0.004	0.11 ± 0.01	0.09 ± 0.01
*K*_10_/min^−1^	0.32 ± 0.05	0.24 ± 0.08	0.30 ± 0.18
*K*_12_/min^−1^	0.26 ± 0.04	0.38 ± 0.01	0.28 ± 0.11
AUC/mg·kg^−1 ^min^−1^	7.56 ± 1.02	37.15 ± 3.06	81.25 ± 15.3
CL(s)/mg·mL^−1^, mg kg^−1^	0.66 ± 0.05	0.27 ± 0.02	0.018 ± 0.02

## Abbreviations

DPDFT: Di-phenyl-di-(2,4-difluobenzohydroxamato)tin(IV)
I.S.: Internal standard
HPLC: High performance liquid chromatography
QC: Quality control
S/N: The signal-to-noise ratio
LOQ: The limit of quantitation
LOD: The limits of detection
RE: Relative error
Cobs: Observed concentration
Cnom: Nominal concentration
SD: Standard deviation
RSD: Relative standard deviation
*t*_1/2*a*_: Distribution half-life
*t*_1/2*β*_: Elimination half-life
AUC: Area under the plasma concentration-time curve
*K*_21_: First order transfer from compartment 2 to compartment 1
*K*_10_: First order elimination from compartment 1
*K*_12_: First order transfer from compartment 1 to compartment 2
Cl: Clearance.
